# Maternal perceptions of vaccinating boys against human papillomavirus (HPV) in Seoul, South Korea: A descriptive exploratory qualitative study

**DOI:** 10.1371/journal.pone.0282811

**Published:** 2023-03-10

**Authors:** Jihye Choi, Christine Markham, Irene Tamí-Maury, Sooyoun Kim, Paula Cuccaro

**Affiliations:** 1 Department of Health Promotion and Behavioral Sciences, University of Texas Health Science Center at Houston, Houston, Texas, United States of America; 2 Department of Epidemiology, Human Genetics and Environmental Sciences, University of Texas Health Science Center at Houston, Houston, Texas, United States of America; 3 Institute of Health and Environment, Seoul National University, Seoul, South Korea; Regional Health Care and Social Agency of Lodi, ITALY

## Abstract

Human Papillomavirus (HPV) vaccination is of paramount importance to reduce HPV-associated cancers in both genders. In South Korea, the prophylactic vaccine is recognized as prevention of cervical cancer with little attention given to male HPV vaccination. The purpose of this study was to explore perceptions of male HPV vaccination and underlying factors for vaccine hesitancy among mothers of unvaccinated boys in Seoul, Korea using a qualitative method. We used a purposive sampling strategy to recruit mothers of unvaccinated middle school-aged boys living in one of the 25 districts in Seoul, supplemented by a snowball sampling approach. We conducted one-on-one telephone interviews with ten mothers using a semi-structured interview guide. Questions probed mothers’ views on vaccinating boys against HPV and the reasons for not vaccinating their sons. We found that mothers were hesitant to vaccinate their sons against HPV due to high out-of-pocket costs, fear of side effects concerning the young age of their sons, and low awareness of HPV and HPV vaccine, all of which stemmed from the exclusion of male HPV vaccination in the national immunization program. Sociocultural factors, including vaccination norms, lack of HPV education, and values associated with sexually transmitted infections were also likely to negatively impact mothers’ vaccination decision-making. Despite the barriers, mothers were willing to accept HPV vaccination when it was framed as cancer prevention for not only the sons but also their future spouses. In conclusion, reasons for Korean mothers’ hesitancy for their sons’ HPV vaccine uptake were multifaceted. Healthcare providers’ role in emphasizing and conveying the importance of gender-neutral HPV vaccination will be essential to alleviate negative sentiments around the vaccine for boys and reduce their risk of compromised sexual health. As an effective public health strategy, tailored cancer prevention messages should be delivered heightening significant benefits of the HPV vaccine beyond the prevention of cervical cancer.

## Introduction

Parental hesitancy about childhood and adolescent immunizations has been a perennial challenge and a global health issue [1, 2]. The complexity of parents’ decision-making and concomitant apprehension becomes amplified if the vaccine of interest is for diseases associated with sexual behaviors. Human papillomavirus (HPV), which is one of the most common sexually transmitted infections (STI), causes approximately 30% of all infectious agent-related cancers [3]; however, HPV-associated cancers are preventable with timely vaccination. HPV vaccine is effective for preventing anogenital cancers in women [4–6]; it has also been approved for administration to males to prevent oropharyngeal, penile, and anal cancers in men [4, 5]. However, parents still seem hesitant about vaccinating their sons [6]. About 4% of boys worldwide had received the full course of the vaccine in 2019 compared to 15% of girls [7] and this drastic gender disparity is ascribed to the absence of gender-neutral HPV vaccination in many parts of the world, especially in Asian countries [8].

In South Korea, the male prevalence of noncervical HPV-associated cancers is rapidly increasing. Korea has a higher prevalence of oropharyngeal, anal, and laryngeal cancers in men than the male prevalence of these cancers in all of Asia combined [9]. However, HPV vaccination in Korea is predominantly recognized as a female-centered concern solely to eliminate cervical cancer [8]. There is a paucity of research on male HPV vaccination and factors underlying HPV vaccine hesitancy among parents of boys in Korea. Recent studies reported that the HPV vaccination rate among a sample of Korean male adolescents was extremely low, ranging from 0.7–1.3% [10, 11] compared to 35.0–43.8% for female adolescents [12]. These findings suggest that parents may have little awareness of their sons’ eligibility for the vaccine or may be disinclined to vaccinate their sons against HPV for different reasons. Bias about male HPV vaccination being viewed as optional and of low priority may also function to validate parental hesitancy, deferral, or non-acceptance [13]. Before implementing policies to promote HPV vaccination in males in Korea, it is important to first understand parents’ initial reactions, individual beliefs, and cultural connotations associated with vaccinating boys against HPV, because parents are primary decision makers for their children’s health [14, 15]. This preliminary work can serve as a starting point to determine contextually appropriate approaches to educate parents about the importance of HPV vaccination for their sons.

Little qualitative research has investigated the knowledge, perceptions, or attitudes of Korean parents towards vaccinating boys against HPV. A previous review on HPV vaccination in males confirmed the need for more qualitative studies to understand parents’ views regarding HPV vaccine uptake for boys in the Asian region [16]. In this study, we targeted mothers, as they tend to be more confident in performing the parental role and more communicative with their children than fathers, and fathers are less likely to participate in research related to children’s vaccination in Korea [17, 18]. Therefore, the present study uses a qualitative approach to explore perceptions of HPV vaccination and reasons for vaccine hesitancy among mothers of unvaccinated boys in Seoul, Korea.

## Methods

### Study design

This study is a secondary qualitative data analysis of an original nationwide research project on adolescent sexual health and rights initiatives in South Korea. We used a descriptive exploratory qualitative approach, which is useful for summarizing and understanding an area of interest [19], and allows for the contextualization of the topic. The study follows the Standards for Reporting Qualitative Research (SRQR) guidelines [20] for qualitative research studies (S1 File).

### Setting and participants

A purposive sampling strategy was used initially to recruit survey participants from an online community of mothers in each of the districts in Seoul, the capital city of Korea. These online communities are interactive channels in which mothers share concerns about raising and educating children [21, 22]. Recruitment announcements were posted on the “announcements” board of the websites. To find additional eligible interviewees, we also used a snowball sampling approach. We asked the participants who were already recruited to recommend other contacts who fit the eligibility criteria and might be potentially willing to be interviewed. Participants were eligible to participate in the study if they were mothers of middle school-aged male adolescents (the equivalent of 7^th^– 9^th^ grades in the United States) who had not received any doses of the HPV vaccine at the time of the interview, lived in one of the 25 districts in Seoul, completed an initial baseline survey and consented to be re-contacted. The study protocol was approved by the Institutional Review Board at Seoul National University (IRB No. 2106/003-013). Written informed consent was obtained from each participant prior to the interview. Each participant was compensated with a $30 e-gift card for their participation in the study.

### Inclusivity in global research

Additional information regarding the ethical, cultural, and scientific considerations specific to inclusivity in global research is included in S1 Checklist.

### Data collection

One-on-one semi-structured interviews were conducted via telephone due to COVID-19 restrictions. The interviews were conducted between August and December 2021 at a time convenient for the participants and lasted an average of 45 minutes The interviews were conducted by the first author, who was experienced in qualitative research and remained neutral and nonjudgmental. The interview guide used in this study was developed based on available literature [23–25] that addressed conceptual frameworks of the health belief model and the socio-ecological model. The main questions asked in the referenced studies were: 1) *What is your view on vaccinating boys against HPV*? 2) *What are the reasons for not vaccinating your son*? Validated interview questions designed for parents of daughters were adapted by replacing daughters with sons. The interview guide for the present study included 10 open-ended questions concerning mothers’ attitudes and beliefs about HPV and HPV vaccination. Mothers were asked to offer their thoughts regarding the HPV vaccine, their opinion on vaccinating boys against HPV, and their reason for delaying or refusing HPV vaccination for their sons. We continued to interview participants until data saturation was reached at 10 interviews, whereby no new codes or themes were identified. All interviews were conducted in the Korean language and audio recorded. The recordings were transcribed verbatim and the transcripts were spot-checked to ensure quality control.

### Data analysis

Data were analyzed manually by the first author using thematic analysis, which is considered an apposite method of analysis for seeking to understand experiences, thoughts, or behaviors across a dataset [26, 27]. An initial codebook was developed that includes codes based on prominent features across the entire dataset from the preliminary reading of the transcripts. Transcripts were then coded with multiple codes being allocated where necessary, and emerging codes were added to the codebook (S2 File). Codes were analyzed for patterns and used to identify an emergent thematic framework comprising a hierarchy of meaningful themes and sub-themes of each individual theme. Codes and themes were reviewed by the first author, and names were generated for each theme in English following verification. Salient text passages were extracted to illustrate the emergent themes and were translated into English.

## Results

### Characteristics of the sample

An overview of the characteristics of the participants is presented in Table 1. We interviewed a total of 10 mothers who were recruited using purposive and snowball sampling strategies. The average age of the mothers was 44.2 years and the majority of the mothers were university graduates. None of the mothers had been previously diagnosed with HPV-related diseases and six mothers had never received an HPV vaccine. Six of the participating mothers had a daughter and the average age of the mothers’ sons was 14.1 years.

**Table 1:** Characteristics of the interviewed mothers participating in the study (N = 10).

Participant characteristics	N
Age (mean age: 44.2 years)	
< 45 years	6
≥45 years	4
Educational level	
High school	1
University	7
Graduate school	2
Previous HPV diagnosis	
Yes	0
No	10
Vaccination status	
Fully vaccinated	4
Partially vaccinated	0
Never vaccinated	6
Son’s grade in middle school (mean age: 14.1 years)	
1^st^ grade	3
2^nd^ grade	5
3^rd^ grade	2
Mother has a daughter	
Yes	6
No	4

### Major themes

We identified three major themes: 1) hesitancy towards HPV vaccine for boys due to the absence of gender-neutral HPV vaccination; 2) cultural, social, and familial influences regarding HPV vaccine for boys; and 3) cancer prevention framing increases acceptance of HPV vaccine for boys. These themes and sub-themes are listed in Table 2 are discussed in detail below with illustrative quotations from participants.

**Table 2:** Major themes and sub-themes.

Theme	Sub-theme
Hesitancy towards HPV vaccine for boys due to the absence of gender-neutral HPV vaccination	High out-of-pocket costs of HPV vaccine Fear of side effects and concerns about the young age Lack of HPV and HPV vaccine knowledge
Cultural, social, and familial influences regarding HPV vaccine for boys	Providers do not actively recommend the vaccine for boys School-based sex education does not cover STIs Other mothers have not vaccinated their sons Sexual health communication rarely happens at home
Cancer prevention framing increases acceptance of HPV vaccine for boys	Perceived severity of reproductive cancers for sons and future partners increases HPV vaccine acceptance

#### Theme 1. Hesitancy towards HPV vaccine for boys due to the absence of gender-neutral HPV vaccination

Mothers were overall hesitant about giving their sons the HPV vaccine due to out-of-pocket costs, concerns about vaccine safety, and lack of vaccine knowledge. These barriers were associated with the exclusion of the vaccine for males from the national immunization program (NIP) in Korea. Mothers were reluctant to pay for the HPV vaccine for their sons, especially when it was not listed as a mandatory childhood immunization. Mothers understood the vaccine as optional and collectively expressed that they would be willing to consider the vaccine if it were offered for free or at affordable prices. For example, one mother explained:

*I would be more than willing to get my son vaccinated if it were free or cheaper*. *Right now*, *the vaccine is too expensive*, *and it is only recommended*, *it is not really mandatory for boys*. *I don*’*t really see the point of getting it any sooner*.

(Mother J, 50 years old, mother of a 16-year-old son and a daughter)

Moreover, mothers were concerned about the safety of the vaccine and its side effects for their young sons. Mothers believed that, unlike routine female vaccination, insufficient data to support male HPV vaccination, especially for young boys, may be the reason for its current exclusion from the NIP. While mothers acknowledged no signs of side effects from previous HPV vaccination of their daughters, they were still apprehensive because the vaccine was not readily available for males. For example, one mother of a vaccinated daughter noted:

*Even though I didn*’*t see any side effects when my daughter got the vaccine*, *just the fact that male HPV vaccination is not part of the national immunization program makes me worry about potential side effects for my son such as infertility when he gets married*.

(Mother D, 48 years old, mother of a 15-year-old son and a daughter)

Another mother noted the rapidly changing patterns of sexual behavior in younger generations and the need for vaccination against STIs in early adolescence. However, she did not seem to be able to relate it to her own son, believing that he was too young for the vaccine with potential risk. This discrepancy is illustrated by the following quotation:

*I was surprised about the eligible HPV vaccination age*. *I understand that age at first sex is decreasing nowadays*, *and so early prevention of sexually transmitted infections is important*. *But I still think that my son is too young to receive this vaccine associated with sex*. *He just started middle school this year*.

(Mother G, 40 years old, mother of a 12-year-old son)

In addition, the vast majority of the mothers had heard of male HPV vaccination only in vague terms and had very limited knowledge about specific details, such as “how old boys have to be to get the vaccine, how many times they have to get the vaccine or benefits of the vaccine that outweigh the risks” (Mother A, 48 years old, mother of a 15-year-old son and a daughter). The lack of knowledge about HPV was more pronounced among mothers who did not have a daughter and had fewer opportunities to be advised about HPV immunization. Many of them did not even know that males could get the HPV vaccine, let alone the fact that males are potential carriers of STIs. As a result, they questioned the necessity of vaccinating their sons, thinking that the vaccine was solely for cervical cancer prevention. For example, one mother said:

*I don*’*t think it is absolutely necessary for my son to get the HPV vaccine that prevents cervical cancer when he doesn*’*t even have a cervix… I assume it is not an urgent matter*. *People don*’*t generally recognize that males are carriers of sexually transmitted infections anyway*.

(Mother C, 42 years old, mother of a 14-year-old son)

Mothers described that part of their lack of knowledge was attributed to how the vaccine was labeled. In Korea, the English abbreviation of “HPV” and the Korean word for human papillomavirus are unfamiliar to lay people and thus rarely used. The HPV vaccine is commonly referred to as “the cervical cancer vaccine” and mothers of sons only are particularly unfamiliar with the vaccine. The misleading name of the vaccine has given mothers a false message that the vaccine is only for preventing female cervical cancer and is irrelevant to their sons. The name of the vaccine has inadvertently created confusion among parents of boys. For example, one mother (Mother H, 43 years old, mother of a 14-year-old son and a daughter) reported: “I have always thought that it was only for women because of the name and I am starting to hear that it’s not. I am still not convinced. I’m just confused.” Another mother worried that changing the name of the vaccine to the STI vaccine without reference to cervical cancer may connote sexual promiscuity, raising stronger aversion to the HPV vaccine among parents, even after establishing vaccine efficacy for boys.

*This vaccine is still called the cervical cancer vaccine today*, *so parents are often asking*, *my son doesn*’*t have a cervix*, *so why does he need the vaccine*? *But then labeling this a “sex-related” vaccine or anything that sounds sexual would immediately cause negative reactions from parents*. *I think we are just so unfamiliar with the vaccine*.

(Mother F, 41 years old, mother of a 13-year-old son and a daughter)

In general, the absence of gender-neutral HPV vaccination produced substantial barriers for mothers including the high cost of the vaccine, skepticism about vaccine safety specifically for boys, and the persistent belief that HPV vaccination is optional for boys or less important. Little knowledge among mothers about male susceptibility to HPV and their eligibility for the HPV vaccine was another major hindrance to HPV vaccine uptake among boys. Furthermore, the HPV vaccine being established as the cervical cancer vaccine in Korea did little to improve mothers’ knowledge about and apprehension towards male HPV vaccination.

#### Theme 2. Cultural, social, and familial influences regarding the HPV vaccine for boys

Several external factors deterred mothers from considering HPV vaccination for their sons. There was a consensus among mothers that healthcare providers did not proactively recommend the HPV vaccine for boys. One mother stated (Mother D, 48 years old, mother of a 15-year-old son and a daughter): “I am not decided whether I should vaccinate my son against HPV, mainly because I have not heard anything from our family doctor yet.” One mother of a vaccinated daughter described that decision to vaccinate her son would depend on her family doctor’s response (Mother F, 41 years old, mother of a 13-year-old son and a daughter): “If our doctor approves and recommends an optimal time for vaccination, I may decide to vaccinate him. It’s just that until now no one recommended the vaccine for my son.” Mothers also often encountered the HPV vaccine and its effectiveness for boys through media rather than through healthcare providers. For example, one mother said (Mother A, 48 years old, mother of a 15-year-old son): “I didn’t get any information directly from our pediatrician, but I knew a little about the vaccine from a medical article I once came across and advertisements from drug companies.” Another mother added (Mother H, 43 years old, mother of a 14-year-old son and a daughter): “I heard from social media that not only females but males are also eligible for the vaccine. That was my only source of information, I have not heard anything from health clinics.”

Mothers mentioned that the current sex education that adolescents receive in school still did not cover information regarding STIs and the prevention of STIs via vaccination. One mother of an only son (Mother C, 42 years old, mother of a 14-year-old son) pointed out that her lack of knowledge about male HPV vaccination was partly due to limited sex education, implying this relatively new concept should have been introduced by sexual health educators: “HPV vaccination for men and boys is so new to us and we have little knowledge about it because it has never been included as part of the sex education curriculum at school.” Mothers were under the impression that education about STI prevention may be of less importance for males, which served as another barrier to their decision to vaccinate their sons. For example, one mother discussed:

*I feel that sex education at school is really important to educate kids about sexually transmitted infections and the need for vaccination for both genders*. *As far as I know*, *sex education still does not cover that*. *Then it makes us think that it*’*s okay for the kids to not know about prevention of HPV or other sexually transmitted infections*. *I was surprised that nothing has changed since our days*. *It*’*s the same old curriculum*.

(Mother I, 43 years old, mother of a 14-year-old son)

Another factor that influenced mothers’ vaccine decision-making was other parents’ perceptions of the HPV vaccine for sons. With the ongoing tendency among community members not to vaccinate their sons, mothers were not willing to deviate from the norm of non-vaccination and experiment on their own sons. When queried about other mothers of boys and their perceived beliefs and practices regarding the vaccine, one mother described that her peers in the community were mostly unaware of their sons’ eligibility for HPV vaccination.

*I would say more than 70% of mothers of boys did not know about HPV vaccination at all*. *Maybe 20% only heard of it before*, *and less than 10% of the mothers actually knew what it was*. *I think I want to wait and see what other mothers of boys do*. *If they are accepting the vaccine*, *then I might be tempted to do the same*. *I don*’*t need to experiment when it is common not to vaccinate sons*.

(Mother B, 47 years old, mother of a 16-year-old son and a daughter)

In general, mothers were reticent to openly discuss the sexual health of their sons with other parents given the sensitivity of the subject. For example, one mother remarked:

*I have never heard other mothers of sons talk about this vaccine*, *and I don*’*t know of anyone who has vaccinated their son against HPV*. *We rarely discuss boys*’ *sexual health*. *I think we are being cautious*. *We don*’*t want others to think that my son or myself we are too interested in sex-related matters*.

(Mother E, 40 years old, mother of a 12-year-old son)

Many mothers expressed that sexual health communication with their sons rarely occurred at home due to the mothers’ discomfort with the topic. The cultural influence of circumventing the sensitive topic and inexperience talking about sex for the mothers themselves led to feelings of diffidence, which impeded effective mother-son sexual health communication. One mother reflected:

*I have never said a single word about sex with my parents or friends when I was growing up*, *so it is only natural that I feel uncomfortable having a conversation about my son*’*s sexual health*. *To this day*, *we have not really talked about it with our son at home mainly because I find it embarrassing*. *I think I will put it off to my husband because he might be better and more understanding*.

(Mother J, 50 years old, mother of a 16-year-old son and a daughter)

In addition to their feelings of embarrassment, some mothers noted significant difficulty in responding to questions their sons asked about sexual health. Mothers’ willingness to explain HPV and HPV vaccination waned even more when faced with such challenges. Consequently, their role and involvement in sexual health communication at home were minimal. One mother explained:

*I understand that my son can get curious about sex*. *There are things that he asks me*, *but I am not exactly sure how I should respond to that*. *That*’*s why I don*’*t see myself ever educating my son about HPV and HPV vaccination*. *My son asks our family doctor more often than he asks me or my husband because it is comfortable for him that way*.

(Mother B, 47 years old, mother of a 16-year-old son and a daughter)

Overall, mothers were less inclined to accept male HPV vaccination largely due to the discomfort around the vaccine associated with sex and their feelings of embarrassment. Despite some media coverage about the HPV vaccine for males, mothers would forgo or postpone vaccinating their sons due to lack of recommendations from healthcare providers, limited sex education at school, perceptions of other mothers, and little mother-son communication about sexual health.

#### Theme 3. Cancer prevention framing increases acceptance of HPV vaccine for boys

Despite the limited uptake of the HPV vaccine in in Korean males due to various barriers, mothers’ acceptability of the vaccine for boys increased when the concept was framed as cancer prevention. Protection from STIs was perceived as less relevant to mothers of young boys, but the association of the vaccine with the prevention of cancers exerted a greater impact on maternal perspectives on the vaccine. For example, one mother explained:

*I don*’*t think we as mothers necessarily connect it to the prevention of sexually transmitted infections for our sons quite yet*. *But*, *when I heard it can prevent several cancers for males*, *not just cervical cancer*, *I didn*’*t want to ignore it*. *It*’*s much easier to accept the vaccine if we think of it as a preventive measure for cancer*, *regardless of the kind of cancer*. *If there is a vaccine that can prevent at least one kind of cancer*, *I agree that my son should get it*. *I mean who doesn*’*t want their children to be protected from cancer*?

(Mother F, 41 years old, mother of a 13-year-old son)

In addition, mothers who had a daughter mentioned the risk of their son’s future partner developing cancer if their son remained unvaccinated. Messages about cervical cancer prevention for female partners of sons seemed to strongly resonate among these mothers. The fact that their son’s vaccine uptake can prevent cancer for not just himself but also for his significant other contributed to vaccine acceptability. For example, one mother said:

*I heard that my son*’*s vaccine uptake can also prevent cancer for my son*’*s future partner*. *If not getting the HPV vaccine on my son*’*s part could put his future wife at risk of developing cancer*, *then I think it*’*s important to get him vaccinated in the near future*. *I just don*’*t want anything bad to happen to my son and his wife*, *both of their health matters*.

(Mother B, 47 years old, mother of a 16-year-old son and a daughter)

The therapeutic effect of the HPV vaccine in preventing STIs did not appeal to mothers as a reason to vaccinate their sons most likely due to their young age. However, mothers were willing to accept HPV vaccination for their sons when it was framed as cancer prevention, regardless of the type of cancer. The fact it can prevent reproductive cancers for males convinced mothers to think that HPV vaccination was relevant for their sons. Mothers’ intention to vaccinate their sons against HPV also increased upon the realization that male vaccination can protect their son’s future spouses from HPV-associated cancers.

## Discussion

### Principal findings

In the present study, we conducted semi-structured one-on-one interviews with a purposive sample of ten mothers of unvaccinated sons living in the capital city of Korea. This qualitative study sought to understand mothers’ current knowledge and beliefs regarding HPV vaccination in males and factors that underlie their hesitancy towards vaccinating boys against HPV. We found that suboptimal levels of HPV and vaccine knowledge among mothers were primarily due to the feminization of HPV and the HPV vaccination policy in Korea that excludes males. Traditionally, the long-standing connection of HPV to cervical cancers has overshadowed the importance of preventing other non-cervical HPV-associated cancers [4]. This disconnect was evident in our study as many of the mothers did not realize their sons’ eligibility for the vaccine due to female-centered discourse around HPV that neglects the risk of associated diseases for both genders. A qualitative study on Korean American parents’ vaccination beliefs also documented this gender bias as the parents believed the vaccine was beneficial only for daughters and were reluctant to learn more about the vaccine since they had only sons [28]. Moreover, the avoidance of the unfamiliar English term “HPV” and the medical jargon in the Korean language has unintentionally misguided lay people to refer to the vaccine as “the cervical cancer vaccine.” This label has instilled an erroneous idea in mothers that it does not apply to their sons. However, despite the existing misalignment of the vaccine with females [4] and current knowledge deficits, mothers’ willingness to vaccinate increased when it was framed as cancer prevention for their sons and their future spouses. This finding reinforces the notion that individuals are more likely to undergo a behavioral change when they have higher perceived susceptibility or when an issue becomes more salient to them [29]. It also demonstrates that composing messages that appeal to protecting children against cancer is key to increasing HPV vaccine acceptance [30]. For example, messages about penile cancer prevention may draw attention from parents of boys and result in increased vaccine acceptability [31]. Focus on perceived relevance is vital when promoting HPV vaccination in males, and cancer protection benefits should be presented for males along with their future partners using pertinent gain-framed messages.

Although HPV is not a gender-specific infection, the absence of publicly funded HPV vaccination for males has engendered negative sentiments in mothers of sons about the safety and high out-of-pocket costs of the vaccine. Mothers assumed that exclusion of male HPV vaccination from the NIP and lack of recommendation by providers might be due to insufficient research behind it, which has been reported as one of the reasons for parental decisions not to vaccinate sons [15, 32, 33]. While being receptive to their daughters’ uptake because female vaccination is routinized and strongly recommended via formal invitation, mothers in this study expressed fear of unknown side effects for boys, especially considering their young age for a vaccine associated with STIs, as reported in a previous study [34]. Similarly, Chinese immigrant parents had ambivalent perceptions and doubted the claimed protection period for the HPV vaccine if immunized when young [35]. It could be that mothers did not want to tolerate unknown risk and vaccinate their sons for an effect needed many years later when their sexual debut has occurred [35]. Mothers were also hesitant about the immense cost to vaccinate their sons against what was perceived as a female disease. However, they were willing to consider the vaccine if it were provided for free or at cheaper prices, which aligns with a previous study conducted in Canada where mothers were moderately willing to get their sons’ HPV vaccine for free, but much less willing to pay the out-of-pocket cost [36]. This sentiment is likely common among financially underprivileged parents [37]. Recent studies in Canada, Ghana, Nigeria, and Sweden have underscored that high cost would be a major barrier to being interested in or receiving STI vaccines and that those that require multiple doses or boosters, such as the HPV vaccine, will have greater financial implications for public acceptance of out-of-pocket costs [37–40]. A Greek study also found that having a low income was associated with lower HPV vaccination acceptance among mothers for their sons, although income did not influence women’s attitudes for themselves and their daughters [41]. Implementation of gender-neutral HPV vaccination is critical to mitigating these challenges, as it can promote routinization of male HPV vaccination and guard against any misinformation thereby alleviating vaccine hesitancy in mothers of boys [42].

We found that sociocultural factors play a substantial role in shaping maternal perceptions of male HPV vaccination and their vaccine decision-making. In Asian cultures, parents tend to be heavily influenced by, or even defer decision-making regarding HPV vaccination to physicians [43]. We observed this cultural attribute in our study. Lack of recommendation from providers and perceived disapproval from the mothers’ most trusted source of health information was a reason for them to be undecided or postpone the vaccine for their sons. Similarly, providers’ recommendations affected HPV vaccination decisions of Korean American parents [28] due to the cultural value of hierarchical authority in which a doctor is viewed as highly esteemed [28]. Given the parents’ adherence to expert medical opinion inherent in Asian culture, public health authorities and health care providers should proactively instill the importance of vaccinating boys against HPV until this information permeates parents’ social networks. Mothers in this study also reported that very few others in their community had heard of the HPV vaccine for boys and believed that other mothers chose not to vaccinate their sons. Other parents’ resistance to HPV vaccination for boys appeared to give more weight to mothers’ decisions against the vaccine that they perceived to carry potential risks. This phenomenon implies that individuals more readily accede to perceptions of others’ vaccination beliefs and practices, known as descriptive social norms [44], when the vaccine of interest is relatively new [45]. Our finding confirms that of previous research on English- and Spanish-speaking parents in the United States that beliefs about other parents’ practices with regard to HPV vaccine for sons are a potentially powerful factor [46]. Parental beliefs that their peers are not vaccinating their sons can develop less interest in vaccinating their sons, driving further skepticism about the HPV vaccine [46]. Such attitudes can also be delineated in terms of social-network theory where non-vaccinators tend to know and associate with other non-vaccinators [47]. Furthermore, the taboo nature of sexual communication in Korea [28], and efforts to conform to such societal norms can explain mothers’ low proclivity to discuss the sexual health of their sons with other mothers. Mothers are likely to postpone or refuse the HPV vaccine for boys due to these cultural values in conjunction with current vaccination norms. To mitigate these issues, community-based interventions that entail a casual environment for vaccination promotion and the correction of misinformation may change parents’ perceptions of the HPV vaccine for boys. For example, public service announcements and webinars in health clinics to raise awareness about male HPV vaccination as well as images of positive parenting to protect their son against HPV on media have been reported as effective methods [28, 48, 49].

Our data indicate that mother-son communication about sexual health let alone prevention of STIs is minimal, which is common in Asian countries where premarital sex is traditionally discouraged [50]. Asian parents tend to approach these issues indirectly or implicitly, avoiding words in their language for sexual health topics, such as HPV [51]. Evidence from the United States suggests that parent-child sexual health communication can curb negative sexual health outcomes [52] and improve HPV vaccination rates [53]. The consistent discomfort mothers felt to initiate conversations with their sons about sexual health may have contributed to their lack of confidence or refusal to talk about HPV vaccination. This could, in part, be explained by the omission of HPV from school-based sexual education, which can misguide mothers to think that it is not necessary for their sons to know about HPV vaccination or to continue the discussion at home. Mothers in this study, most of whom were university graduates, hesitated to vaccinate their sons but also believed that the topic of HPV vaccination should be part of the sex education their sons receive at school. It is interesting to note that contrary to our findings, mothers with a lower level of education in a Greek study were reported to be less likely to accept the HPV vaccine for their children than those with higher education [41]. Researchers from Australia, Sweden, and the United States have recommended that education about HPV should ideally be provided through schooling using age-appropriate, updated resources to emphasize that males have an equal burden of HPV infections [54–56]. This effort may facilitate mother-son sexual health communication at home with a more positive outlook on HPV vaccination for sons.

### Strengths and limitations

We conducted the interviews via telephone due to the COVID-19 pandemic, resulting in loss of nonverbal data. However, telephone interviews allowed visual anonymity for the interviewees, who were able to share thoughts more comfortably and give honest responses about this sensitive subject. While none of the mothers had previously been diagnosed with HPV-related disease, which poses a risk of bias, it was entirely unintended as previous diagnosis of HPV was not one of the eligibility criteria. The study excluded parents of partially or fully vaccinated sons, but it is an inherent issue in this context as HPV vaccination for boys is very rare in Korea. Another limitation is the representation of parents through only mothers. However, fathers are less likely to communicate with their adolescents and mothers are typically the parent that communicates sexual health information to their children [57, 58]. Although only ten mothers were interviewed, the same set of questions were asked of all mothers in the purposive sample with a certain degree of participant homogeneity, which led to the collection of rich data and early attainment of thematic saturation; therefore, the sample size was considered appropriate. Strengths of this study include the use of an inductive approach within thematic content analysis that helped generate new insights with detailed accounts of the data. Most importantly, this study has provided in-depth insights into Korean mothers’ perspectives on male HPV vaccination as little qualitative research has previously investigated Asian mothers’ response to vaccinating boys against HPV using their own words.

## Conclusions

This qualitative study explored the perceptions of male HPV vaccination among mothers of adolescent sons in Korea. Themes gleaned from our data highlight that the reasons for mothers’ hesitancy towards HPV vaccine uptake for their sons are multifaceted, primarily stemming from the exclusion of male HPV vaccination from the national immunization program. The absence of gender-neutral HPV vaccination has ingrained in parents misconceptions about HPV vaccination such as the vaccine being unnecessary for males. Sociocultural factors, including vaccination norms, education, and cultural values associated with sexual health are also likely to exacerbate mothers’ hesitancy to vaccinate their sons. To alleviate negative sentiments around the HPV vaccine for boys and reduce their risk of compromised sexual health, pediatric and adolescent healthcare providers in community-based clinics should be at the forefront to emphasize and convey the importance of gender-neutral HPV vaccination. Finally, tailored cancer prevention messages should be delivered to parents of boys heightening significant benefits of the vaccine beyond the prevention of cervical cancer.

## Supporting information

S1 File Standards for Reporting Qualitative Research (SRQR)*. (DOCX)

S2 File Codebook. (DOCX)

S1 Checklist Inclusivity in global research. (DOCX)
