# Supplementary material for: Maternal perceptions of vaccinating boys against human papillomavirus (HPV) in Seoul, South Korea: A descriptive exploratory qualitative study
Source: PLoS One. 2023 Mar 10;18(3):e0282811. doi: 10.1371/journal.pone.0282811 (PMC10004501; doi:10.1371/journal.pone.0282811)
Supplement: S2 File — (DOCX) [file pone.0282811.s002.docx]

Codebook

**Theme 1. Hesitancy towards the HPV vaccine for boys due to the absence of gender-neutral HPV vaccination**

1. High cost
2. Fear of side effects (too young)
3. Lack of knowledge (did not know that boys can also get the vaccine; my son does not have a cervix so why does he need the vaccine?)
   - Need to change the name of the vaccine
   - More information is needed (dose/schedule, benefits of the vaccine, STIs)

**Theme 2. Cultural, social and familial influences regarding the HPV vaccine for boys**

1. Providers did not recommend (information from media but
2. School-based sex education is not effective (no improvement in the curriculum, does not cover HPV or STIs)
3. Other mothers have not vaccinated their sons
4. Sex communication rarely happens at home (don’t feel comfortable explaining the vaccine to son)

**Theme 3. Framing HPV vaccination as cancer prevention increases acceptance of the HPV vaccine for boys**

1. Cancer prevention is key (focus is on the fact that it prevents cancer is key)
2. Perceived threat of HPV related diseases for son (without the vaccine, son may be at higher risk of contracting the virus, partner’s health, problems after marriage)

| **Code** | **Description** | **Example** |
| --- | --- | --- |
| Fear of side effects |  | The only concern I have is side effects and that would really hinder my decision.  I am aware that she is eligible for the vaccine and that the vaccine has a lot of benefits but I feel that there is still insufficient follow-up evidence on this vaccine. I am cautious to vaccinate my son so early in his adolescence.  I am still quite hesitant and worried about the safety of this vaccine and its potential side effects.  I could not find clear evidence regarding the effectiveness and benefits of the HPV vaccine for boys so I am a bit hesitant.  Even though I didn’t have see any side effects when my daughter was vaccinated, just the fact that male HPV vaccination is not part of the national immunization program makes me worry about potential side effects for my son such as infertility when he gets married. |
| High cost |  | But the thing is that it is out-of-pocket for boys. The cost is about $150 per dose, which means $300 for the full series. I am hesitant about the cost.  I would get my son vaccinated if boys are offered the vaccine free of cost.  Cost is always an issue. I don’t really feel that my son needs the vaccine at this moment and so high cost is definitely a barrier.  I do find the cost of the vaccine for boys a little bit burdensome  I would be more than willing to get my son vaccinated if it were free or cheaper. Right now, the vaccine is too expensive, and it is only recommended, it is not really mandatory.  I have heard about male HPV vaccination before but since it is not a mandatory immunization I am not sure it if it is worth the money when it is so expensive.  I would certainly hope for a cheaper price. I would get my son vaccinated if the vaccine were offered at no cost. |
| Little knowledge; Unfamiliarity with the HPV vaccine for boys |  | All I knew was that boys were eligible for the HPV vaccine but I didn’t know when to vaccinate my son or how often.  No one informed me about by what age my son should be vaccinated and the benefits of the vaccine.  I don’t think we know too well about the benefits of the HPV vaccine. We are just told to vaccinate them  All I know about the HPV vaccine is that it is highly recommended to women because it can prevent cervical cancer. |
| Name of the vaccine |  | I have always thought that it was only for women because of the name and I am starting to hear that it’s not. I am still not convinced. I’m just confused.  Right now, the name of the vaccine is definitely a problem.  This vaccine is still called the cervical cancer vaccine today, so parents are often asking, my son doesn’t have a cervix, so why does he need the vaccine? But then labeling this a “sex-related” vaccine or anything that sounds sexual would immediately cause negative reactions from parents. I think we are just so unfamiliar with the vaccine. |
| HPV vaccine is not necessary |  | I questioned if it is really necessary at this point because it is not an urgent matter. Even in health clinics, I have never been recommended the vaccine for my son.  I don’t think it is absolutely necessary for my son to get the HPV vaccine that prevents cervical cancer when he doesn’t even have a cervix and I assume it is not an urgent matter. People don’t generally recognize that males are the carriers of the sexually transmitted infections anyway.  I still wonder if it is necessary for my son to get the vaccine to prevent cervical cancer when he doesn’t even have a cervix. |
| Son is too young to get a vaccine associated with STI |  | I was surprised about the eligible HPV vaccination age. I understand that age at first sex is decreasing nowadays, and so early prevention of sexually transmitted infections is important. But I still think that my son is too young to receive this vaccine associated with sex. He just started middle school this year.  9-year-old boys are still growing and we should be cautious about what medication or vaccination we give them.  More than that, I think he is too young to get a vaccine that is associated with sex. |
| Providers did not recommend |  | I am not decided whether I should vaccinate my son against HPV, mainly because I have not heard anything from our family doctor yet.  I might ask again and if our doctor approves and recommends an optimal time for vaccination, I may decide to vaccinate him. It is just that until now no one has recommended the vaccine for my son.  Even in health clinics, I have never been recommended the vaccine for my son. |
| Get information from media over health providers |  | I heard from social media that not only females but males are also eligible for the vaccine. That was my only source of information, I have not heard anything from health clinics.  I didn’t get any information directly from pediatrician, but I knew a little about the vaccine from a medical article I once came across and other advertisements from drug companies. |
| Other mothers have not vaccinated their sons |  | I have never heard other mothers of sons talk about this vaccine, and I don’t know of anyone who has vaccinated their son against HPV. We rarely discuss boys’ sexual health. I think we are being cautious. We don’t want others to think that my son or myself we are too interested in sex-related matters.  When I asked other parents of boys, I would say more than 70% of mothers of boys did not know about HPV vaccination at all. Maybe 20% only heard of it before, and less than 10% of the mothers actually knew what it was. I think I want to wait and see what other mothers of boys do. If they are accepting the vaccine, then I might be tempted to do the same. I don’t need to experiment when it is common not to vaccinate sons. |
| Rarely ever talk about it with other mothers |  | It is true that we rarely ever talk about sex and sexual health of boys.  We don’t discuss boys sexual health as much. We rarely do. I think we are being cautious. We don’t want others to think that my son or myself we are too interested in sex-related matters. |
| Sex communication at home (rarely happens at home) mother feels uncomfortable |  | I find it a little embarrassing to talk about this kind of topic I feel that I do nothing to relieve his curiosity.  We don’t freely talk about sex at home, in fact, I don’t think we have ever had a sex-related conversation with our son.  Normally, I would not say that we are open to talk about sex at home. I am not sure yet but all we are aware is that our son’s body is changing slowly but he is too young to know about reproductive health.  I have never said a single word about sex with my parents or friends when I was growing up, so it is only natural that I feel uncomfortable having a conversation about my son’s sexual health. To this day, we have not really talked about it with our son at home mainly because I find it embarrassing. I think I will put it off to my husband because he might be better and more understanding.  I feel that it would be more appropriate and comfortable for my son to talk about it with his dad and my husband would be more understanding. |
| Not sure how to respond to son (when he asks)  Hard to explain the vaccine to children |  | I understand that my son can get curious about sex. There are things that he asks me, but I am not exactly sure how I should respond to that. That’s why I don’t see myself ever educating my son about HPV and HPV vaccination. My son asks our family doctor more often than he asks me or my husband because it is comfortable for him that way.  I find it a little embarrassing to talk about this kind of topic I feel that I do nothing to relieve his curiosity.  But for most of them, they are just being dragged and getting the vaccine not being educated.  I didn’t explain to my daughter what the vaccine was for exactly, I just told her it is something like the flu vaccine. |
| School sex education is ineffective and does not include STI/HPV  No progress |  | I would trust health providers too but we seek their advice only when we are ill so I feel that school is more important. But unfortunately, we don’t communicate with health educators as much and I am not really sure about their role either.  There are fewer male health educators and I do think that having an equal sex ratio of health educators may help to educate boys about why they need the HPV vaccine.  After several sessions of sexual education from school, he was complaining why they were teaching the same thing over and over when kids all know about it by now.  I feel that sex education at school is really important to educate kids about sexually transmitted infections and the need for vaccination for both genders. As far as I know, sex education still does not cover that. Then it makes us think that it’s okay for the kids to not know about prevention of HPV or other sexually transmitted infections. I was surprised that nothing has changed since our days. It’s the same old curriculum.  HPV vaccination for men and boys is so new to us and we have little knowledge because it has never been included as part of sex education curriculum at school.  As far as I know, there is no difference between elementary school and middle school regarding sex-education curriculum. |
| Cancer prevention |  | I don’t think we as mothers necessarily connect it to the prevention of sexually transmitted infections for our sons quite yet. But, when I heard it can prevent several cancers for males, not just cervical cancer, I didn’t want to ignore it. It’s much easier to accept the vaccine if we think of it as a preventive measure for cancer, regardless of the kind of cancer. It there is a vaccine that can prevent at least one kind of cancer, I agree that my son should get it. I mean who doesn’t want their children to be protected from cancer?  It there is a vaccine that can prevent at least one type of cancer, I should get it. |
| Family support |  | No one in my family would disagree, my husband would agree if we were told that it is necessary for our son.  I don’t think any friends or relatives of ours would oppose our son receiving the vaccine. |
| Son’s health in the long term |  | At first, I was doubtful about the vaccine and did not see its necessity or importance. But the more I think about it, if it can be a potential issue when he gets married, then I might as well get him vaccinated.  I heard that this vaccine can protect male sexual health and prevent cancer for my son’s partner. So I have been thinking about getting my son vaccinated some time in the near future.  But I do worry about my son’s sexual health. For girls, it is more obvious the aftermath of sexual intercourse but for boys, there is no way of knowing. They could become more promiscuous or they could be equally hurt just like girls.  However, I do think that if I ever hear about sex-related health issues in other boys, my son can get it too. |
| Worried that not getting the vaccine can be a potential issue when he gets married (partner’s health) |  | I have a daughter so just when I was thinking about getting her vaccinated, I also heard from the media that not only females but males are also eligible for the vaccine. So I have been thinking whether or not to vaccinate my son too. I heard that by getting my son vaccinated against HPV, it can protect male sexual health and prevent cancer for my son’s partner.  If not getting the vaccine would have a health implication on my son’s partner in later years then I think it is reasonable to get him vaccinated.  I heard that my son’s vaccine uptake can also prevent cancer for my son’s future partner. If not getting the HPV vaccine on my son’s part could put his future wife at risk of developing cancer, then I think it’s important to get him vaccinated in the near future. I just don’t want anything bad to happen to my son and his wife, both of their health matters.  Without the vaccine, they are at higher risk of contracting the virus so I guess if it is to prevent that, then I agree with boys getting HPV vaccination. |
| Need more information – Benefits of the vaccine |  | I would like to know more about the HPV vaccine. For example, how old boys have to be to get the vaccine, how many times they have to get the vaccine or benefits of the vaccine that outweigh the risks, and to what extent is this vaccine effective? I am just worried what if my son takes risky behaviors knowing that he is already vaccinated against HPV.  Over-trusting this vaccine worries me and that’s why I want to know for those who are vaccinated, how effective was it, for example what percentage? I am not even sure if there are resources that I can use to acquire that information. |
| Need more information – dosage and schedule |  | My decision will depend on how much information I get regarding the vaccine dosage and schedule. |
| Need more information – STIs |  | I feel that if people are given more information about specific cases of STI prevention from this vaccine then, they would buy it more. We are sporadically exposed to news media or stories about STIs but we don’t talk about it in reality.  I want to know specifically the effect of vaccination on reduction of STIs in greater detail. It would be more meaningful to people if they were given numerical values as to how much the incidence of STIs decreased as a result of vaccination. For example, it would be helpful to see a direct comparison between the cost to treat cervical cancer or other HPV related cancers and the cost to receive the vaccine (additional information like this). |
| Vaccine hesitant |  | I would also trust the HPV vaccine more if it is part of the mandatory vaccine list. |
